# Cumulative oesophageal dose and risk of high-grade toxicity in thoracic re-irradiation: a dose/toxicity analysis

**DOI:** 10.1016/j.ctro.2026.101108

**Published:** 2026-01-09

**Authors:** Robert Rulach, Stephen Harrow, Anthony J. Chalmers, John Fenwick

**Affiliations:** a University of Oxford, Department of Oncology, Old Road Campus Research Building, Roosevelt Drive, Oxford OX3 7DQ, United Kingdom; b NHS Lothian, Edinburgh Cancer Centre, Western General Hospital, Crewe Rd S, Edinburgh EH4 2XU, United Kingdom; c School of Cancer Sciences, University of Glasgow, Glasgow G61 1QH, United Kingdom; d University College London, Department of Medical Physics & Biomedical Engineering, Gower St, London WC1E 6BT, United Kingdom

**Keywords:** Re-irradiation, Intra-thoracic recurrence, Non-small cell lung cancer, Oesophageal cancer

## Abstract

**Background and purpose:**

Re-irradiation (re-RT) in recurrent thoracic cancer is being performed more often and can cause severe toxicity. Information is lacking on safe re-irradiation dose constraints. We modelled the relationship between cumulative oesophageal dose and grade 3 or worse (≥G3) oesophageal toxicity to develop dose constraints.

**Materials and methods:**

We performed a literature search for reports of thoracic re-RT and selected studies that quoted cumulative oesophageal maximum dose (cD_max_) in equivalent dose in 2 Gy fractions (EQD2) and ≥G3 toxicity. Additional collected data were inter-treatment interval and concurrent chemotherapy use (conCT) at re-irradiation. Logistic regression analyses were performed. The models were used to predict the cD_max_ associated with a 5% ≥G3 toxicity rate. Model performance was assessed using the Pearson correlation coefficient (PCC).

**Results:**

We identified 21 studies (505 patients), with 49 ≥G3 toxic events. The median oesophageal cD_max_ and interval were 84.8 Gy_3_ (3.7 – 220.6 Gy_3_ EQD2) and 15.5 months (1–162) respectively. Use of conCT and oesophageal cD_max_ were significantly associated with toxicity on univariable and multivariable modelling (both p < 0.001). The maximum likelihood doses associated with 5% risk of ≥G3 toxicity with/without chemotherapy were cD_max_ 43.0 Gy_3_ (95% CI: −18.5, 108.8) and 94.2 Gy_3_ (95% CI: 79.6, 142.8) respectively. The model had a PCC of 0.75 (p = 0.013) suggesting good correlation to the dataset.

**Conclusion:**

The models predict a 5% toxicity at cD_max_ 43.0 Gy_3_ and 94.2 Gy_3_ EQD2 with/without chemotherapy. This supports a cD_max_ constraint of <95 Gy_3_ EQD2 to limit ≥G3 toxicity to under 5%, consistent with the American Radium Society constraints (<100 Gy_3_ EQD2). ConCT with re-irradiation has a large radiosensitising effect. Limitations of this study include the use of old, retrospective data resulting in wide CIs around the predictions and insufficient data to predict a volumetric constraint. Further modelling with more detailed dose data is required to refine and validate these predictions.

## Highlights

• Thoracic re-irradiation can cause high-grade oesophageal toxicity and cumulative dose constraints have limited evidence.
• Using multivariable logistic regression, we developed a model to predict grade ≥3 oesophageal toxicity from re-irradiation.
• The significant variables were cumulative oesophageal D_max_ and the use of concurrent chemotherapy.
• The model predicted 5% risk of grade ≥3 toxicity at a cumulative oesophageal D_max_ of 94.2Gy_3_ EQD2 (with no chemotherapy).
• This is consistent with current guidelines and may inform re-irradiation planning, patient counselling or future studies.

## Introduction

Thoracic re-irradiation for recurrent lung and oesophageal cancer is delivered with increasing frequency, due to advances in technology, improved survival from initial treatment, and better surveillance post-primary treatment [1], [2]. While there is growing data to suggest that radical re-irradiation has promising efficacy, there remain significant uncertainties in how to safely re-irradiate patients [3], [4], [5]. One key issue is the lack of evidence based cumulative dose constraints. Oesophageal toxicity from primary lung radiotherapy is a significant dose limiting toxicity with recent data suggesting grade ≥2 and grade ≥3 toxicity rates of 48% and 2.2% respectively [6]. For lung re-irradiation, the reported risk of high grade toxicity (i.e. requiring a feeding tube) ranges between 0–9%, with a mean incidence of 2% [7]. The rate of oesophageal fistula/perforation in patients with re-irradiated oesophageal cancer is approximately 20% [8]. The cumulative dose constraint is important to determine as it may influence the re-irradiation dose to the planning target volume (PTV) that can be safely delivered and therefore the likelihood of tumour control.

There is limited data to develop a cumulative dose constraint for the oesophagus. There are no pre-clinical studies that detail the recovery kinetics of the oesophagus. There is little prospective trial data, although there are several retrospective reviews that detail re-treatment doses, albeit using different techniques to calculate accumulated dose, and quoting different dose metrics. Re-irradiation dose and subsequent toxicity has been difficult to predict due to the small numbers in each study.

Several groups have suggested dose constraints based on clinical experience [1], [9], [10], [11], [12]. These groups suggest limiting the cumulative dose to around 100–110 Gy_3_ equivalent dose in 2-Gray fractions (EQD2) although are unable to provide rates or severity of toxicity at that dose. Re-irradiation dose/toxicity models would be useful in providing a risk of toxicity for a given cumulative dose to the oesophagus, as this would allow better counselling of the patient of the likely side-effects from re-irradiation and give clinical teams a target dose constraint for planning purposes.

We collected dose and toxicity data from studies of patients who had re-irradiation to the thorax. We performed logistic regression analyses to determine the relationship between cumulative oesophageal dose and grade 3 or above (≥G3) toxicity. This data was used to develop cumulative dose constraints.

## Materials and methods

### Patients and data

A literature search was conducted using MEDLINE and the Glasgow University search engine, identifying any English language studies from 1st January 1970 to 1st October 2020 which included adult humans who had two courses of radiotherapy for malignancy. The minimum criteria for inclusion were both the cumulative oesophageal dose and the toxicity encountered were published or could be reasonably derived. Animal models were excluded.

**Fig. 1:**
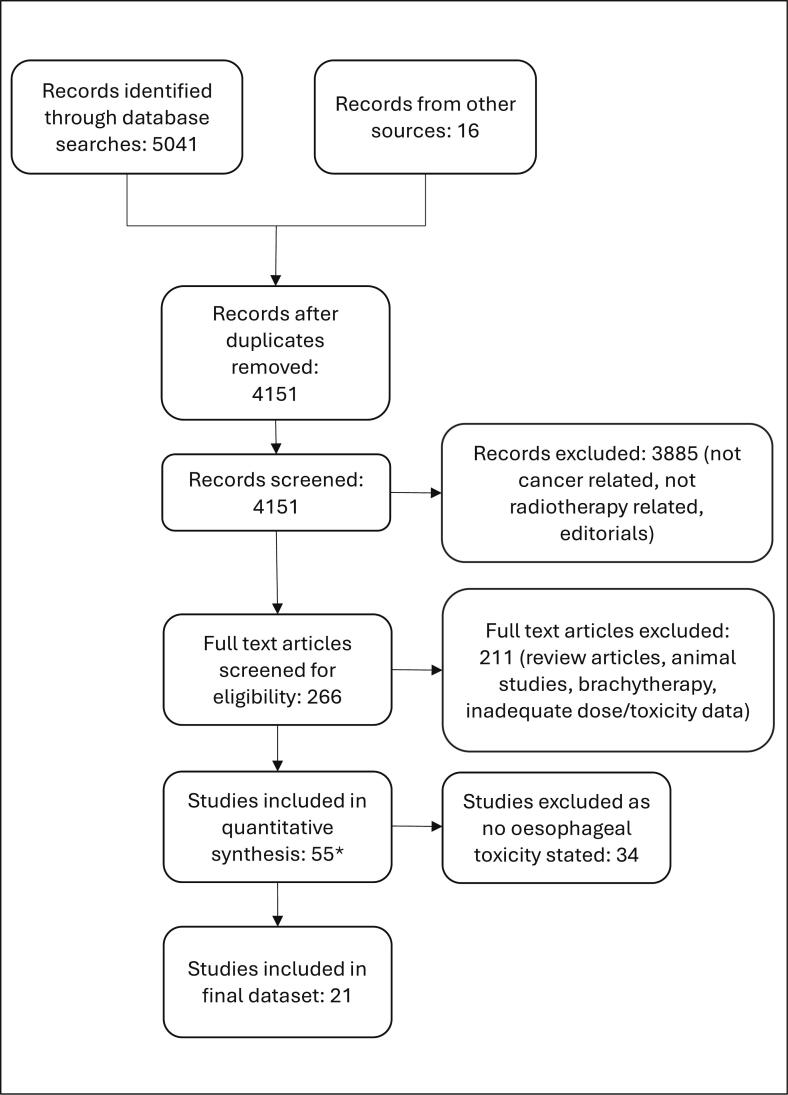
PRISMA diagram.

The MEDLINE search strategy aimed to identify studies that detailed outcomes from patients who had a significant cumulative oesophageal dose (i.e. recurrent oesophageal or recurrent lung cancer). For patients with oesophageal cancer re-irradiation the following search terms were used: (Oesophag* OR Esophag*) AND (retreatment OR re-treatment OR re-irradiation OR reirradiation). For patients with recurrent lung cancer, the terms used were ((lung AND cancer) OR non-small cell lung cancer) AND (retreatment OR re-treatment OR re-irradiation OR reirradiation) AND (dose constraints OR toxicit*). The abstracts of the studies identified were reviewed and were selected for further analysis if they met the minimum inclusion criteria. Pertinent additional papers were also analysed from the selected studies’ references. The PRISMA diagram in Fig. 1 outlines the process to identify the data used for modelling.

Cumulative oesophageal D_max_ (cD_max_), interval, concurrent chemotherapy (conCT), and grade of toxicity observed were collected from the studies. Studies were excluded if they did not state a cumulative oesophageal dose metric or a toxicity rate.

The type of oesophageal toxicities that were grouped as ≥G3 oesophageal toxicity included oesophageal perforation, tracheoesophageal fistula, radiation oesophagitis, dysphagia, oesophageal haemorrhage and stenosis. The type of toxicity grading system used for each study was recorded, to assess if they were comparable. Toxicities within 3 months of re-irradiation were labelled acute, and late if after 3 months. For patients with acute toxicities, an α/β of 10 was used for EQD2 calculations for oesophageal dose, and an α/β of 3 for late toxicity.

Data structure was variable between papers, with some papers presenting grouped data, and other papers presenting patient-by-patient dose information. In studies where the cumulative dose was not directly quoted, the doses from the first and second course of radiation was converted into EQD2, using an α/β ratio of 3. This data was only included in the dataset if the cD_max_ could be reasonably assumed by this method i.e. in locally recurrent oesophageal cancer, where the PTVs would have overlapped and the cumulative PTV dose (quoted in the study) would also equal the dose to the oesophagus.

### Analysis

The modelling process is outlined in Appendix Fig. 1. No imputation was performed for missing data. The potential predictor variables (dose, chemotherapy and interval) were split by their median values and evaluated using $\chi^{2}$ or Fisher’s exact test as suggested in a recent primer on radiotherapy modelling [13].

Logistic regression modelling was conducted on the dataset using the maximum likelihood estimation method. Univariable models were generated for each predictor. Univariable models which had a p-value <0.2 were included in multivariable models.

Leave-one out cross validation (LOOCV) by cohort was used to determine the model that best fitted the data [14]. The data of one study was removed, and a model was fitted to the remaining dataset. This model was then applied to the removed study. The accuracy of each putative model fit from the cohorts with a dataset removed was determined by calculating a log-likelihood score. This calculated (in a model distribution analogous to the model fitting technique) the difference between the predicted toxicity rate and the observed toxicity rate. The log-likelihood scores for each different cohort were summed. The model with the lowest log-likelihood score implied a better fit to data and was chosen to make predictions.

The models were plotted and used to predict the maximum-likelihood 5% ≥G3 oesophageal toxicity. A binomial 95% confidence interval (CI) was not added to the graphs as this would be misleading and would not account for the heterogeneity between studies (e.g. the difference in follow-up duration). The oesophageal dataset was block-bootstrapped 2000 times. Block-bootstrapping (where bootstrapped datasets were generated by selecting study cohorts with replacement) was chosen as this accounted for data overdispersion. Bootstrapped samples were used to calculate a cumulative dose prediction for the 5% and 20% toxicity rates. The lowest 2.5% and top 2.5% predictions were excluded to give the block-bootstrapped 95% CI on the dose leading to the specified toxicity rate.

Assessment of model fit was performed by splitting the collected dataset into deciles. The model predictions (using variables from the collected dataset) of the rate of toxicity by decile were plotted against the observed rate of toxicity for the corresponding decile. This was assessed using a Pearson correlation coefficient.

All analyses were performed using R version 3.6.1 (R Core Team (2013). R: A language and environment for statistical computing. R Foundation for Statistical Computing, Vienna, Austria). A p-value of <0.05 was used for significance for all tests.

## Results

We identified 21 studies suitable for analysis, presenting data collected between 1996 and 2017. There were 505 patients included, with 49 ≥G3 toxic events. The median cumulative oesophageal dose was 84.8 Gy_3_ EQD2 (3.7–220.6 Gy_3_) and the median interval between the courses of radiotherapy was 15.5 months (1–162). The included studies are detailed in Table 1. The dose accumulation methods for each study are summarised in the Appendix Table 1.

**Table 1:** List of studies used to form the oesophageal dataset. The total number of patients included is 505, from 21 studies. 3D-CRT: three-dimensional conformal radiotherapy, BED: biologically equivalent dose, cD_max_: cumulative oesophageal maximal dose, EQD2: equivalent dose in 2 Gray fractions, Fr: fractions, IMPT: intensity modulated proton therapy, IMRT: intensity modulated radiotherapy, NR: not recorded, NSCLC: non-small cell lung cancer, RBE: relative biological effectiveness, Re-RT: re-irradiation, SABR: stereotactic ablative radiotherapy.

Paper	n	Tumour type re-irradiated	Treatment span	Re-treatment Technique	Median cD_max_	Interval (months, range)	Chemo % rate with re-RT	Any Grade 3–5 toxicity (%)	F/u post re-RT (months, range)
Poltinnikov [15]	9	NSCLC	1999–2003	3D-CRT	82.2 (66.8–96.3)	13 (2–39)	33.3	0	5.5 (2.5–30) (f/u to death)
Yamaguchi [16]	12	Oesophagus	1996–2008	3D-CRT	93.8 (87.0–98.9)	8.5 (4–162)	100	50	4 (1–108)
Kim [17]	10	Oesophagus	2007–2011	3D-CRT	98.7 (93.8–111.5)	15.6 (4.8–36.4)	30	30	4.9 (2.6–11.4)
Katano [18]	4	Oesophagus	2011–2016	3D-CRT	88.2 (71.9–97.9)	17.4 (6.4–59.2)	83.3	16.7	8.8 (1–30.4)
Hong [5]	39	Oesophagus	2000–2014	IMRT 56.4%, 3D-CRT 43.6%	112 (80–140)	16 (3–168)	50	7.7	87 (2––206)
Zhou [19]	55	Oesophagus	2003–2012	IMRT or 3D-CRT	115.2 (NR)	12 (6–56)	NR	20	20 (8–70)
Chen [20]	36	Oesophagus	1996–2005	IMRT	99.2 (99.2–107.7)	14.6 (4.5–165)	100	52.8	62 (8–192) (whole group including surgery patients)
Kennedy [21]	21	NSCLC	2008–2017	SABR	18.7 (4.9–37.2)	23 (7–52)	0	0	24 (3–60)
Schlampp [22]	62	NSCLC	2010–2015	IMRT	89.9 (NR)	14 (3–103)	3.2	4.8	8.2 (0–27)
Schroder [23]	30	NSCLC	2011–2017	SABR	81.0 (70.2–103.8)	14 (2–184)	0	0	13 (1–45)
Meijneke [24]	8	NSCLC	2005–2012	90% SABR, 10% Conventional	85.2 (70.5–123.2)	17 (2–33)	0	0	12 (2–52)
Owen [25]	18	NSCLC	2006–2012	SABR	62.5 (38.9–78.4)	18.4 (1.5–112.8)	0	0	21.2 (3.4–50.2)
Kilburn [26]	33	NSCLC	2001–2012	SABR in 91%	69 (11–129)	18 (6–61)	0	3	17 (NR)
Sumita [27]	21	NSCLC	2007–2014	Conventional 90%, Proton 10%	73 (NR)	26.8 (11.4–92.3)	5	0	22.1 (2.3–56.4)
Binkley [28]	38	NSCLC	2008–2014	SABR 73.7%, Conventional 26.3%	44.1 (3.7–220.6)	16 (1–71)	23.7	2.6	17 (3–57)
Maranzano [29]	18	NSCLC or mets	2003–2013	SABR	45 (4–138)	18 (6–90)	0	0	57 (6–132)
Ho [30]	27	NSCLC	2011–2016	Proton (IMPT)	84.8 (57.1–121)	29.5 (0.1–212.3)	48	0	11.2 (2.4–48.5)
Hong [31]	31	NSCLC	2005–2016	IMRT 67.7%, SABR 32.3%	74.4 (NR)	15.1 (4.4–56.3)	9.7	0	17.4 (4.8–76.8)
Ogawa [32]	31	NSCLC or mets	2004–2017	SABR	19.4 (0.8–146.8	NR	0	0	26 (5.5–111)
Griffioen [33]	1	NSCLC	2004–2013	Conventional	120	62	NR	100	6
McAvoy [34]	1	NSCLC	2006–2011	Protons	135.7	36	NR	100	29

The lowest ≥G3 toxicity was seen at a cD_max_ of 60.4 Gy_3_ EQD2 with an interval of 4 months. The type of ≥G3 events are subcategorised in Table 2. There were 57 events in total, but when grouped together, this number reduced to 49 as eight patients had both early and late toxicity. The variables were split by their median values. Factors significantly influencing toxicity rates were interval between treatments, use of concurrent chemotherapy and cD_max_ (Appendix Table 2).

**Table 2:** Type of Grade 3 or above toxicity. G*x*: grade of toxicity at *x* level, NR: not recorded.

	Number	Acute			Late			Type of toxicity	Grading scale
		G3	G4	G5	G3	G4	G5		
Yamaguchi [16]	12	3	1	0	2	0	1	Acute: oesophageal perforation G3 (2), radiation oesophagitis (G3 (1), oesophageal perforation G4 (1). Late: oesophageal stenosis G3 (1), oesophageal perforation G3 (1), oesophageal haemorrhage G5 (1)	CTCAE v3
Kim [17]	10	0	0	3	0	0	0	Acute: oesophageal perforation and trache-oesophageal fistula G5 (3)	CTCAE v3
McAvoy [34]	1	NR	NR	NR	0	1	0	Late: trache-oesophageal fistula G4 (1)	CTCAE v4
Griffioen [33]	1	NR	NR	NR	1	0	0	Late: radiation oesophagitis G3 (1)	CTCAE v4
Kilburn [26]	1	NR	NR	NR	0	0	1	Late: oesophageal haemorrhage G5 (1)	CTCAE v4
Binkley [28]	38	NR	NR	NR	1	0	0	Late: radiation oesophagitis G3 (1)	CTCAE v4
Katano [18]	4	0	0	0	1	0	0	Late: dysphagia G3 (1)	CTCAE v4
Hong [5]	39	0	0	0	3	0	0	Late: trache-oesophageal fistula G3 (3)	CTCAE v4
Zhou [19]	55	NR	NR	NR	0	0	11	Late: oesophageal perforation and trache-oesophageal fistula G5 (11)	CTCAE v3
Chen [20]	36	19	NR	NR	NR	7	NR	Acute: radiation oesophagitis/dysphagia G3 (19) Late: oesophageal haemorrhage/tracheoesophageal fistula G4 (7)	Not recorded
Schlampp [22]	62	NR	NR	NR	1	1	0	Late: oesophageal stenosis G3 (1), tracheoesophageal fistula G4 (1)	CTCAE v4
Total (%)		22 (38.6)	1 (1.8)	3 (5.3)	9 (15.8)	9 (15.8)	13 (22.8)	Perforation/TOF: 30 Oesophagitis/dysphagia: 23 Bleeding: 2 Stenosis: 2	

### Dose/toxicity oesophageal model

ConCT and cD_max_ effects were statistically significant on both univariable and multivariable logistic regression modelling (p-values <0.001). The interval between treatments was not significant (p = 0.991, Appendix Table 3). Two models were created from the dataset, a multivariable model using oesophageal cD_max_ (in EQD2) and conCT and a univariable model using only the cD_max_. The univariable cD_max_ model was compared to the multivariable model (cD_max_ and conCT) using LOOCV. The log likelihood scores were 153.1 and 144.2 for the univariable model and the multivariable model respectively, indicating that the multivariable model better described the data and therefore was used to make predictions.

The multivariable model expression is:

(1) $$p\left(\geq G3\;toxicity\right)=\frac{1}{1+e^{-(-7.0065\;+\;0.0431CD\;+\;2.2065CC))}}$$

where *CD* = cumulative D_max_ and *CC* = concurrent chemotherapy.

### Multivariable model predictions

The dose predicted to give a 5% ≥G3 toxicity rate without chemotherapy is 94.2 Gy_3_; the block bootstrapped 95% CI is 79.6 to142.8 Gy_3_. The dose prediction for 5% toxicity with chemotherapy is 43.0 Gy_3_ (block bootstrapped 95% CI −18.5 to 108.8 Gy_3_). The predicted doses and bootstrapped 95% CI for 10, 20 and 30% toxicity rates are given in Table 3.

**Table 3:** Summary of multivariable model dose predictions with and without chemotherapy. CI: confidence interval, EQD2 Gy: equivalent dose in 2-Gray fractions, MV: multivariable, UV: univariable.

Toxicity rate for ≥G3 toxicity	Model dose prediction (EQD2 Gy)	95% CI lower limit (EQD2 Gy)	95% CI upper limit (EQD2 Gy)	Block bootstrapped 95% CI lower limit (EQD2 Gy)	Block bootstrapped 95% CI upper limit (EQD2 Gy)
MV model–without concurrent chemotherapy					
5%	94.2	82.1	106.4	79.6	142.8
10%	111.6	98.9	124.3	96.1	174.4
20%	130.4	112.5	148.3	104.5	212.0
30%	142.9	120.3	165.5	109.6	236.7

MV model–with concurrent chemotherapy					
5%	43.0	14.7	71.4	−18.5	108.8
10%	60.4	39.0	81.7	15.0	120.6
20%	79.2	64.3	94.1	51.6	136.0
30%	91.7	79.6	103.8	73.7	149.0

**Fig. 2:**
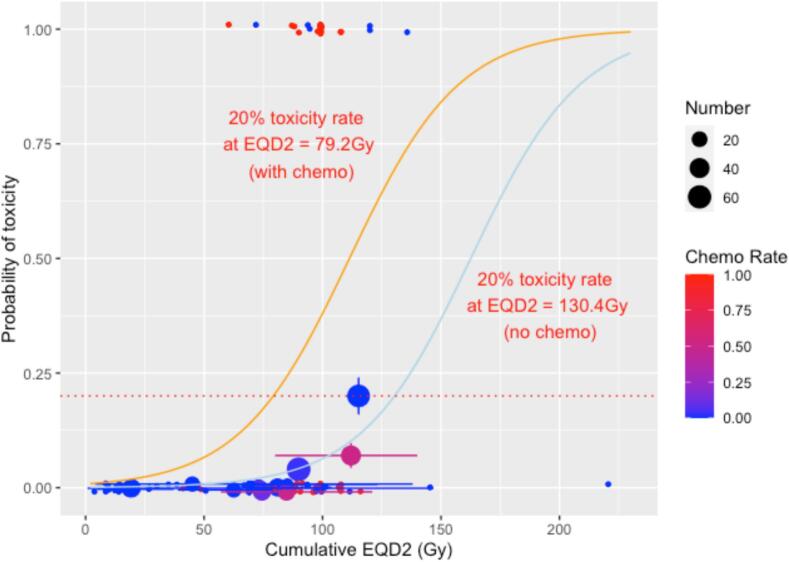
Plot of the oesophageal multivariable models with and without concurrent chemotherapy. The orange line is the fitted regression model with chemotherapy, the light blue line is the fitted regression model without chemotherapy, the red dotted line indicates the 20% toxicity level. The dots represent the toxicity rate from each paper (labelled), with the size of the dots proportional to the number of patients in the study, vertical bars are the 68% binomial confidence interval, horizontal bars represent the range of the doses. The chemotherapy rate for each study is represented on a blue/red scale, with red indicating 100% rate of concurrent chemotherapy, and blue representing a 0% rate. Due to overlapping data points, the scatter plot has been jittered.

The multivariable model plots with and without chemotherapy are plotted in Fig. 2. The dose predicting the 5% risk of toxicity is approximately 40–50 Gy less with chemotherapy than without demonstrating the extent of the radiosensitising effect to normal tissue.

### Validation of the multivariable oesophageal model

**Fig. 3:**
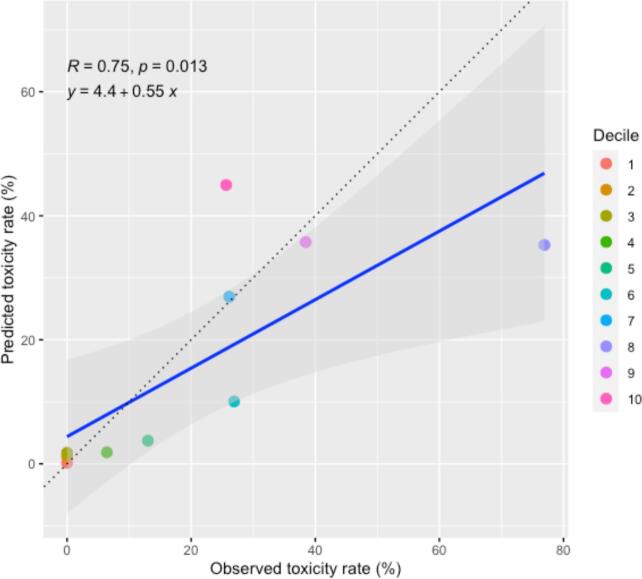
Plot of the actual and predicted oesophageal multivariable model toxicity rates. The blue line is the line of best fit, with the shaded grey area the 95% confidence interval. The black dotted line represents the line of unity. The dots represent the toxicity rate from each decile.

To evaluate the multivariable model, the dataset the model was developed on (i.e. excluding data points that were missing concurrent chemotherapy data) was split into deciles by the modelled risk of toxicity. The cases were not grouped into pre-specified risk bands. The actual and model predicted rate of toxicity was calculated for each decile. For example, the modelled toxicity risk in the 15 cases that made up the first decile was between 0.1 and 0.2%, with no observed toxicity events. The multivariable model had a Pearson correlation coefficient of 0.75 (p = 0.013) suggesting a good correlation between the model predictions and the actual rate of toxicity by decile. This was plotted in Fig. 3, which demonstrates a close correlation between the predicted and observed toxicity deciles up to the 20% toxicity rate. Above this, the confidence intervals become wider, indicating a less good fit to the data.

As an additional validation step, a subset of the dataset that had individual patient level data was used to validate possible constraints. This included 144 patients, with 33 toxic events. Using the maximum-likelihood dose for a 5% ≥G3 toxicity rate (94.2 Gy_3_) from the multivariable model (with no conCT) as a cut-off, the observed rate of toxicity above and below 94.2 Gy_3_ was 46.6% and 7% respectively. This indicated that the cut-off was able to differentiate between high and low toxicity rates and may be useful in a clinical setting.

## Discussion

This is the largest analysis of oesophageal re-irradiation dose data to our knowledge. Oesophageal cD_max_ and conCT were significant in multivariable modelling. Our analysis demonstrated a large risk increase with conCT, indicating that patients having conCT should be counselled for significantly higher risk of toxicity. The model predictions (when split by decile) have a strong correlation (R = 0.75) with the corresponding actual toxicity rate. Therefore, an oesophageal cD_max_ of 94.2 Gy_3_ EQD2 (without chemotherapy) is a reasonable dose constraint aiming to limit the toxicity rate to <5%.

### Source data for the models

The toxicity which was modelled in the models are derived from 11 studies. Six studies presented data from re-irradiation of recurrent oesophageal cancer [[5], [16], [17], [18], [19], [20]]. These studies accounted for 43 (87.8%) of the toxicities in the model. The use of concurrent chemotherapy was common in these patients (median rate 66%, range 0 – 100%). The techniques used were 3D-conformal therapy in three studies, either 3D-CRT or IMRT in two studies and exclusively IMRT in one study. The other five studies were retrospective reviews of re-irradiation in NSCLC which quoted oesophageal dose and toxicity [[22], [28], [33], [34]]. The lung studies accounted for 6 ≥G3 events. The re-irradiation treatment technique was IMRT and SABR in two studies each, and passive scatter proton therapy in one study. The conCT rate was similar to the oesophageal re-treatment group (median 65.8%, range 24–100%). Therefore, the bulk of the toxic events come from re-irradiation of oesophageal primaries rather than lung cancer, albeit using older radiotherapy techniques. This implies that the suggested dose constraint may be too conservative when treating lung cancers.

### Comparison to existing constraints and prospective studies

The suggested dose constraint correlates well with the expert consensus recommendations and contemporary clinical trial data. Most consensus papers suggest a cD_max_ after an interval of a year between treatments of around 100 Gy EQD2 [1], [9], [10], [11], [12]. Due to lack of data, it was not possible to develop a volumetric constraint, although some consensus papers suggest a V60Gy of <40% or V55Gy of <35% [10], [11]. Paradis *et al.* suggested putative recovery rates of 10% between 3–6 months, 25% between 6–12 months and 50% after 1 year. The modelling data indicates that the recovery rate at the median interval of 15 months would be about 35% (assuming that the maximum dose at initial radiotherapy was 70 Gy EQD2). In a recent prospective phase II study in lung re-irradiation, the maximum cumulative oesophageal dose constraint was 100 Gy EQD2 (provided that the interval was greater than 9 months) [35]. Of the 20 patients treated with an oesophageal dose about 80 Gy EQD2, the median dose was 94 Gy (range 80–105 Gy) and there were 8 Grade 1–2 toxic events, and no ≥G3 toxicities observed, indicating that the dose constraint of 94.2 Gy_3_ may be safe in lung re-irradiation.

## Limitations

There are several sources of possible bias in this study. From a methodological approach, we used a systematic literature review with clear inclusion and exclusion criteria. There was one reviewer of the studies and therefore could be a source of bias. However, we feel that the inclusion criteria for the selection of papers were unambiguous, and that a second reviewer was unlikely to change the core dataset. This study uses only published data, and therefore publication bias may be significant. Studies where no toxicity was seen may not be published and without this negative data, the models may give a falsely high rate of toxicity. Additionally, published studies are likely to come from academic centres, which may treat a different population to other radiotherapy centres.

The model predictions should be interpreted with caution, as there are several areas of potential inaccuracy. The CIs are very wide, for example for the 5% toxicity rate, the 95% bootstrapped CI range from 79.6 Gy to 142.7 Gy_3_. This is in part due to the large degree of heterogeneity between the studies, inherent in the process of collecting any retrospective data of a relatively uncommon treatment. The dataset was developed from retrospective studies which were performed over several decades using different radiotherapy techniques. The radiotherapy technique may be outdated, and this may limit generalisability to contemporary clinical practice. Studies came from many different centres, for different tumour groups, with a range of methods of cumulative dose accumulation and follow-up times. Despite this, if an oesophageal re-irradiation dose is less than 79 Gy_3_ (without chemotherapy), then it is highly likely to have a low toxicity rate (<5%). Conversely, given the 95% CI upper limit is 142 Gy_3_ the 94 Gy_3_ constraint may be too conservative. Further data is required to refine these models.

Further sources of inaccuracy with the data include one study combined hyperthermia with re-irradiation [16]. The data from this study were included as the study authors found that hyperthermia had no influence on toxicity. Another study grouped Grade 2–4 toxicity together, therefore a small number of toxic events may be grade 2 rather than ≥G3 events [20]. In some studies, the actual oesophageal dose was not quoted [[17], [18], [19]]. As these studies were relating to oesophageal retreatment, it was assumed that the oesophageal D_max_ is equivalent to the PTV dose. This may underestimate the dose as there may be dosimetric hot spots in the PTV which could make the cD_max_ higher than the prescription dose.

There are some possible confounders. Several papers were included that used 3D conformal radiotherapy, whilst more recent papers used IMRT. Volumes irradiated with the former treatment would be larger, and therefore the risk of toxicity higher. The use of conCT in lung cancer is only in stage III disease (which generally indicated nodal involvement). Nodal involvement would mean that the re-irradiation PTV would likely include the oesophagus. Therefore, the increased toxicity seen with conCT may simply indicate higher stage disease at re-irradiation. Nevertheless, chemotherapy is a known radiosensitiser therefore there is a strong reason for the increased toxicity seen by conCT, but the models did not test if the stage of disease was also significant.

The difference in the tumour type re-irradiated is likely to significantly affect the model predictions. Re-irradiation of the oesophagus is likely to involve a longer part of the oesophagus, the full circumference and to a higher dose compared to the lung re-irradiation plans where the dose and treated oesophageal volume will be minimised. For example, Yamaguchi *et al*. (oesophageal re-irradiation) quoted a median field area of 29  cm^2^ (range 21–40  cm^2^), and Schlampp *et al*. (NSCLC re-irradiation) gave a median length or oesophagus at lung re-irradiation of 1 cm (range 0–17 cm). The presence of an oesophageal tumour may also predispose patients to fistulate or perforate, making re-irradiation toxicity harder to accurately discern. Unfortunately, the volume of oesophagus irradiated (or the mean oesophageal dose or the V60Gy) were rarely quoted in the other studies. This means that the volume of oesophagus, which is likely to be an important factor in toxicity, was unable to be modelled.

The way the cumulative dose was calculated varied depending on the paper. Some papers used rigid or deformable dose registration and determined the cumulative dose using a software-based technique of converting the dose to EQD2. Older studies used no image registration techniques and the EQD2 was calculated manually. The studies reported slightly different dose metrics. Several studies quote dose to 1 cc (D1cc) rather than cD_max_ or used a slightly different α/β ratio. The effect of this would be to underestimate the delivered D_max_.

An important limitation of this study is that early and late toxicity were grouped together. This was done to ensure that any serious toxicity was included in the model and to ensure large enough events for the modelling to be statistically valid. To compensate for the different outcomes, where early toxicity was quoted, the EQD2 was calculated using an α/β of 10 to ensure that the dose and biological effect are congruent. Early Grade 3 toxicity represented 38.5% of the toxicity in the dataset (mainly radiation oesophagitis), with early or late grade 4 and 5 toxicity with 45.6% (mainly perforation or trachea-oesophageal fistula). With no predominant group, it is difficult to use the model to accurately predict the timing of the toxicity. For patients, the difference between early and late toxicity is significant. Early grade 3 oesophageal toxicity (oesophagitis) is likely to recover (although is severe enough to warrant hospitalisation), whereas late oesophageal toxicity (stricture, fistula, perforation) is likely to be permanent.

### Strengths

This large data collection and analysis of cumulative oesophageal doses has identified statistically significant predictors for serious toxicity. This analysis supports current clinical practice based on the consensus dose constraints and allows clinicians to counsel their patients more accurately to the risk of serious oesophageal toxicity.

Additionally, the number of cases that treated patients to high doses was reasonable (152 out of 505 exceeded the dose constraint), which indicates that despite the inherent data challenges, there were enough toxic events to adequately model this relationship. Further robustly collected cumulative dose and toxicity data from the ESTRO-EORTC E2-RADIatE project and the REPAIR study (NCT06558175) will further refine this constraint [36]. Prospective clinical studies are also in progress to also collect more dose data and volumetric information, which should make the dose constraint more precise. Although we have suggested a dose constraint of 94 Gy_3_, it must be noted that re-irradiation is typically a highly individualised treatment. It may be reasonable to exceed this dose where the risk of symptoms of uncontrolled cancer outweighs the risk of re-irradiation, and the model plots will assist in predicting how the risk changes over the suggested constraint.

## Conclusions

The relationship between cumulative dose, conCT and toxicity outlined in this study suggests at 5% risk of serious toxicity at cumulative doses at 94 Gy (no chemotherapy). The use of conCT increases the likelihood of ≥G3 toxicity significantly. This data can be used to counsel patients of the risk or re-irradiation, for re-irradiation planning and for future research in this field such as dose escalation studies. Further volumetric data from prospective studies or robust registries are required to refine the model and improve prediction accuracy.

## CRediT authorship contribution statement

**Robert Rulach:** Conceptualization, Methodology, Investigation, Data curation, Formal analysis, Writing – original draft. **Stephen Harrow:** Conceptualization, Methodology, Resources, Supervision, Writing – review & editing, Funding acquisition. **Anthony J. Chalmers:** Conceptualization, Supervision, Resources, Writing – review & editing, Funding acquisition. **John Fenwick:** Conceptualization, Methodology, Writing – review & editing, Supervision.

## Declaration of competing interest

The authors declare that they have no known competing financial interests or personal relationships that could have appeared to influence the work reported in this paper.
