# Supplementary material for: Cumulative oesophageal dose and risk of high-grade toxicity in thoracic re-irradiation: a dose/toxicity analysis
Source: Clin Transl Radiat Oncol. 2026 Jan 9;57:101108. doi: 10.1016/j.ctro.2026.101108 (PMC12828362; doi:10.1016/j.ctro.2026.101108)
Supplement: Supplementary Data 1 [file mmc1.docx]

**Appendix**

The appendix is divided into two sections:

1. Information with further detail on methodology
2. Information on source data, the results of simple χ^2^ testing of the data split by their median value, and the univariable and multivariable modelling results.

*Methodology*

Search strategy

A literature search was conducted using MEDLINE and the Glasgow University search engine, identifying any English language studies from 1st January 1970 to 1st October 2020 which included adult humans who had two courses of radiotherapy for malignancy, where both the cumulative oesophageal dose and the toxicity encountered were published. Animal models were excluded.

For patients with oesophageal cancer re-irradiation the following search terms were used: (Oesophag* OR Esophag*) AND (retreatment OR re-treatment OR re-irradiation OR reirradiation). For patients with recurrent lung cancer, the terms used were ((lung AND cancer) OR non-small cell lung cancer) AND (retreatment OR re-treatment OR re-irradiation OR reirradiation) AND (dose constraints OR toxicit*).

Assess missing data and test if difference from complete data

Crude testing of difference in toxicity rates (median split, relative risk table)

Univariable logistic regression modelling, proceeding to multivariable models with any predictors with p<0.2

Test potential multivariable model combinations

Of the significant models, use leave one out cross validation to generate a log likelihood score to identify best fitting model

Collected data from literature search

Use best fitting model to make dose predictions, plot model, and predicted/observed plot and assess correlation

Block bootstrap the dataset 2000 times and refit the model to synthetic datasets for bootstrapped 95% CI

Validate the maximum likelihood dose and block bootstrapped doses using the individual patient level data in the collected dataset

Based on the validation results, determine dose constraint for a 5% toxicity rate

**Appendix Figure 1.** Flow chart detailing the process to select a dose constraint

*Source data analysis*

Comment re: missing data

There was a large amount missing data: 340 cases had data missing regarding fractional split and 4 regarding the interval. Patients with missing data had a statistically significant higher mean cumDmax compared to the included data (97.1 vs 69.1, t-test p-value <0.001). There was also a higher rate of toxicity in the group with no missing data with 26.1% compared to 1.8% in the missing data group (Chi sq test p-value <0.001).

**Appendix Table 1.** List of studies used to form the oesophageal dataset.

The total number of patients included is 505, from 21 studies. BED: biologically equivalent dose, DIR: deformable image registration, EQD2: equivalent dose in 2 Gray fractions, Fr: fractions, NR: not recorded, PTV: planning target volume, RBE: relative biological effectiveness, Re-RT: re-irradiation, SABR: stereotactic ablative radiotherapy

| Paper | n | Individual data/Grouped | Initial prescription dose/fr | Re-RT prescription dose/fr | Uncertainty/method of dose accumulation |
| --- | --- | --- | --- | --- | --- |
| Poltinnikov^25^ | 9 | Individual | median 52 (50-66)Gy/assumed 2Gy/fr (estimated) | median 35 (17.5-40)Gy/ median 3.5Gy (3-4) fractions | NSCLC retreats - Assumed median dose for first treatment, calculated dose for second treatment, no direct data for cumulative oesophageal dose. Quoted toxicity is acute, so used the EQD2(10) for this effect, and only for 9 patients due to the other 8 not having significant dose to their oes. No DIR or EQD2 conversion, but merged plans |
| Yamaguchi^13^ | 12 | Individual | median 60 (50.4-70)Gy/median 30 (28-35) fr | median 39 (30-60)Gy/median 24.5 (15-42) fr | Oesophagus retreats - 54.5% of patients also had hyperthermia with re-irradiation (but paper showed that it had no influence on toxicity so included). No DIR or EQD2 conversion. |
| Kim^14^ | 10 | Individual | median 50.4 (50.4-63)Gy/median 28 (27-35) fr | median 50.4 (50.4-63)Gy/median 28 (27-35)fr | Oesophagus retreats - Oes doses not quoted exactly but assumed PTV dose = oes dose (as oesophagus is the target), calculated cumulative dose by adding prescription doses up, no DIR |
| Katano^15^ | 4 | Individual | 50.4Gy/28fr for all | median 45 (30-50.4)Gy/median 25 (21-45) fr | Oesophagus retreats - Oes doses not quoted exactly but assumed PTV dose = oes dose (as oesophagus is the target). No EQD2 conversion, no DIR. |
| Hong^16^ | 39 | Grouped | 74.11 (48–86.32) Gy BED 10 | 60 (25.41–84.87) Gy BED 10 | Oesophagus retreats – no DIR, manual EQD2 calculation. |
| Zhou^17^ | 55 | Grouped | mean 61.2Gy/1.8-2Gy per fr | median 54 (18-66)Gy/1.8-2Gy per fr | Oesophagus retreats - Oes doses not quoted exactly but assumed PTV dose = oes dose (as oesophagus is the target). No DIR, no EQD2 conversion. |
| Chen^18^ | 36 | Grouped | median 54 (54-63)Gy/32 | 50.4Gy/28 fr | Oesophagus retreats - Grouped Grade 2-4 toxicity together. No DIR, no EQD2 conversion. |
| Kennedy^26^ | 21 | Grouped | median 54 (50-54)Gy/ median 3 (3-5) fr | median 50 (50-54)Gy/ median 5 (3-5) fr | NSCLC retreats – used DIR and EQD2. |
| Schlampp^19^ | 62 | Grouped | median 60 (36-70)Gy/median 32 (13-38) fr (estimated ) | median 38.5 (20-60)Gy/median 19 (3-30) fr (estimated) | NSCLC retreats - Used α/β of 4 for EQD2 calculations. No DIR quoted. |
| Schroder^27^ | 30 | Grouped | NR | NR | NSCLC retreats - Uses a subset of the original 42 patients,used rigid then DIR, used EQD2 conversion |
| Meijneke^28^ | 8 | Grouped | median 60 (30-60)Gy/median 3 (1-25)fr (whole group) | median 51 (20-60)Gy/median 5 (3-10) fr (whole group) | NSCLC retreats - Uses a subset of a larger group, used DIR and EQD2 conversion |
| Owen^29^ | 18 | Grouped | median 60 (39-70)Gy/median 30 (12-35) fr | median 50 (40-60)Gy/ median 4 (3-10) fr | NSCLC retreats – rigid registration, some form of EQD2 conversion but not entirely clear how |
| Kilburn^30^ | 33 | Grouped | median 60 (22.5-80.5)Gy/median 30 (1-37)fr | median 50 (20-70.2)Gy/median 10 (1-35) fr | NSCLC retreats – rigid registration, MIM vista for EQD2 conversion |
| Sumita^31^ | 21 | Grouped | EQD2 median 60 (43.1-87.5)Gy (10) | EQD2 median 60 (50-87.5)Gy (10) | NSCLC retreats - Dose to Oesophagus given as D1cc for initial RT and D10cc for re-RT rather than Dmax, rigid registration and EQD2 conversion |
| Binkley^20^ | 38 | Individual | median 50 (20-74)Gy/median 4.5 (1-37)fr | median 50 (20-177.5)Gy/median 4 (1-54) fr (including multiple re-RT courses) | NSCLC retreats – used DIR and EQD2 conversion, quotes EQD2 dose to D1cc therefore Dmax could be higher |
| Maranzano^32^ | 18 | Grouped | 40 (16-60)Gy/5 (2-30)fr | 40 (25-50)Gy/5 fr | Lung or mets to lung – no DIR, calculated EQD2. |
| Ho^33^ | 27 | Grouped | EQD2 median 60 (36-226.8)Gy | EQD2 median 66 (43.2-84)Gy | NSCLC retreats – used Velocity for DIR and EQD2 dose conversion. 22/27 has composite doses |
| Hong^34^ | 31 | Grouped | EQD2 median 66 (43.13-125) Gy | EQD2 median 57.2 (36-110)Gy | NSCLC retreats – unclear dose registration, did do EQD2 conversions |
| Ogawa^35^ | 31 | Grouped | BED median 112.5 Gy (10) (75-119.6) | BED median 105 Gy (10) (64.2-119.6) | Lung or mets to lung.- no details on image registration, but did calculated EQD2 |
| Griffioen^21^ | 1 | Individual | 60Gy/30f | 60Gy/30f | NSCLC retreats – uses rigid registration on Velocity and dose conversion, Quotes dose to D1cc |
| McAvoy^22^ | 1 | Individual | 63Gy/45fr | 39.6(RBE)Gy/22fr | NSCLC retreats – converts to EQD2 but doesn’t describe image registration method |

**Appendix Table 2.** Results of χ2 and Fisher’s exact tests when oesophageal dataset split by median values.

cD_max_: cumulative maximum dose to an organ at risk, G3: grade 3

|  | No ≥G3 toxicity | Any ≥G3 Toxicity (early or late, %) | P-value |
| --- | --- | --- | --- |
| Median cDmax (n=505) |  |  |  |
| <84.8 | 238 | 2 (0.8) |  |
| >84.8 | 218 | 47 (17.7) |  |
|  |  |  | <0.001* |
| Median interval (n=505) |  |  |  |
| <15.5 | 209 | 41 (16.4) |  |
| >15.5 | 247 | 8 (3.1) |  |
|  |  |  | <0.001* |
| Median concurrent chemotherapy rate (n=505) |  |  |  |
| 0 | 245 | 18 (6.8) |  |
| >0 | 211 | 31 (12.8) |  |
|  |  |  | 0.003* |

**Appendix Table 3.** Results from univariable and multivariable modelling.

cD_max_: cumulative maximum dose to an organ at risk

| Predictor | Toxicity | P-value | Number |
| --- | --- | --- | --- |
| Univariable modelling results | | | |
| Interval | Grade ≥3 | 0.991 | 501 |
| Chemotherapy | Grade ≥3 | <0.001* | 505 |
| cD_max_ | Grade ≥3 | <0.001* | 505 |
| Multivariable modelling results | | | |
| Chemotherapy | Grade ≥3 | <0.001* | 505 |
| cD_max_ | Grade ≥3 | <0.001* |  |
